# Potential Industrial Production of a Well-Soluble, Alkaline-Stable, and Anti-Inflammatory Isoflavone Glucoside from 8-Hydroxydaidzein Glucosylated by Recombinant Amylosucrase of *Deinococcus geothermalis*

**DOI:** 10.3390/molecules24122236

**Published:** 2019-06-15

**Authors:** Te-Sheng Chang, Tzi-Yuan Wang, Szu-Yi Yang, Yu-Han Kao, Jiumn-Yih Wu, Chien-Min Chiang

**Affiliations:** 1 Department of Biological Sciences and Technology, National University of Tainan, Tainan 70005, Taiwan; szuyi08231995@gmail.com (S.-Y.Y.); aa0920281529@gmail.com (Y.-H.K.); 2 Biodiversity Research Center, Academia Sinica, Taipei 115, Taiwan; tziyuan@gmail.com; 3 Department of Food Science, National Quemoy University, Kinmen County 892, Taiwan; 4 Department of Biotechnology, Chia Nan University of Pharmacy and Science, No. 60, Sec. 1, Erh-Jen Rd., Jen-Te District, Tainan 71710, Taiwan

**Keywords:** 8-hydroxydaidzein, stable, soluble, anti-inflammation, amylosucrase, *Deinococcus geothermalis*

## Abstract

8-Hydroxydaidzein (8-OHDe), an *ortho*-hydroxylation derivative of soy isoflavone daidzein isolated from some fermented soybean foods, has been demonstrated to possess potent anti-inflammatory activity. However, the isoflavone aglycone is poorly soluble and unstable in alkaline solutions. To improve the aqueous solubility and stability of the functional isoflavone, 8-OHDe was glucosylated with recombinant amylosucrase of *Deinococcus geothermalis* (DgAS) with industrial sucrose, instead of expensive uridine diphosphate-glucose (UDP-glucose). One major product was produced from the biotransformation, and identified as 8-OHDe-7-α-glucoside, based on mass and nuclear magnetic resonance spectral analyses. The aqueous solubility and stability of the isoflavone glucoside were determined, and the results showed that the isoflavone glucoside was almost 4-fold more soluble and more than six-fold higher alkaline-stable than 8-OHDe. In addition, the anti-inflammatory activity of 8-OHDe-7-α-glucoside was also determined by the inhibition of lipopolysaccharide-induced nitric oxide production in RAW 264.7 cells. The results showed that 8-OHDe-7-α-glucoside exhibited significant and dose-dependent inhibition on the production of nitric oxide, with an IC_50_ value of 173.2 µM, which remained 20% of the anti-inflammatory activity of 8-OHDe. In conclusion, the well-soluble and alkaline-stable 8-OHDe-7-α-glucoside produced by recombinant DgAS with a cheap substrate, sucrose, as a sugar donor retains moderate anti-inflammatory activity, and could be used in industrial applications in the future.

## 1. Introduction

Daidzin (D) and genistin (G) are major components of soy isoflavone in soybeans, which are the glucoside derivatives of soy isoflavone aglycone daidzein (De) and genistein (Ge), respectively. De and Ge aglycones have been studied widely and demonstrated multiple bioactivities [1]. In addition to De and Ge, some soy isoflavone derivatives have been isolated from fermented soybean foods, and shown to possess multiple bioactivities [2]. 8-Hydroxydaidzein (8-OHDe), an *ortho*-hydroxylation derivative of De, was first isolated from the fermentation broth of *Streptomyces* sp. in the presence of soybeans [3], and then from many fermented soybean foods [4,5,6,7,8]. In recent decades, 8-OHDe has been evaluated to possess many bioactivities, such as suppression of multi-drug resistance in Caco-2 colon adenocarcinoma cells [9], irreversible inhibition of tyrosinase [10,11], anti-melanogenesis [12,13], inhibition of aldose reductase [14], and anti-inflammation [15,16].

Recently, Seo et al. and Wu et al. developed mass production processes for 8-OHDe from biotransformation of De by *Aspergillus oryzae* [17,18]. The availability of a large quantity of 8-OHDe provides more opportunities for the application of 8-OHDe in the industry. However, although 8-OHDe has many bioactivities, and can be obtained on a large scale, the isoflavone has drawbacks of low solubility and high instability in alkaline solutions [19,20]. These drawbacks limit the applications of 8-OHDe, unless one can improve the half-life of isoflavone with higher solubility and stability.

Biotransformation of natural products by microorganisms and/or enzymes provides a route to improve the properties of the original compounds [21,22]. Among different kinds of flavonoid biotransformation, glycosylation of flavonoids usually holds great promise to increase the solubility of the original compounds. For example, the aqueous solubility of soy isoflavones is improved about 30-fold through glycosylation [23]. Likewise, the glycosyl-biotransformation of 8-OHDe might improve its aqueous solubility and stability. In nature, glycosylation of flavonoids is usually catalyzed with glycosyltransferases (GTs), which use activated uridine diphosphate-glucose (UDP-glucose) as a sugar donor, and transfer the sugar to a flavonoid acceptor [24]. A previous study used the recombinant BsGT110 from *Bacillus subtilis* to catalyze glucosylation of 8-OHDe [20]. The results showed that the aqueous solubility and stability of the isoflavone glucosides (8-OHDe-7-*O*-β-glucoside and 8-OHDe-8-*O*-β-glucoside) were greatly improved. In addition, such biotransformation was not easily scaled up to the industrial level because of the expensive substrate, UDP-glucose.

However, some glycoside-hydroxylases (GHs) also catalyze glucosylation of flavonoids [25,26]. For example, the amylosucrase, GH of the GH13 family, is able to catalyze glucosylation of flavonoids with cheaper sucrose as a sugar donor, which is one-millionth the cost of UDP-glucose [26]. An amylosucrase from *Deinococcus geothermalis* (DgAS) is one of the promising bio-catalysts in glucosylation of phenolic molecules, because of its thermally-stable and higher activity than other amylosucrases [27,28]. In the present study, the DgAS enzyme was produced in recombinant *Escherichia coli,* and the purified DgAS was detected to catalyze the glucosylation of 8-OHDe. The biotransformed glucosidic product was then purified with chromatography, identified with spectrometric methods. The aqueous solubility, stability, and anti-inflammatory assay of the produced isoflavone glucoside were determined. 

## 2. Results

### 2.1. Production of DgAS Protein in Recombinant Escherichia coli

**Figure 1:**
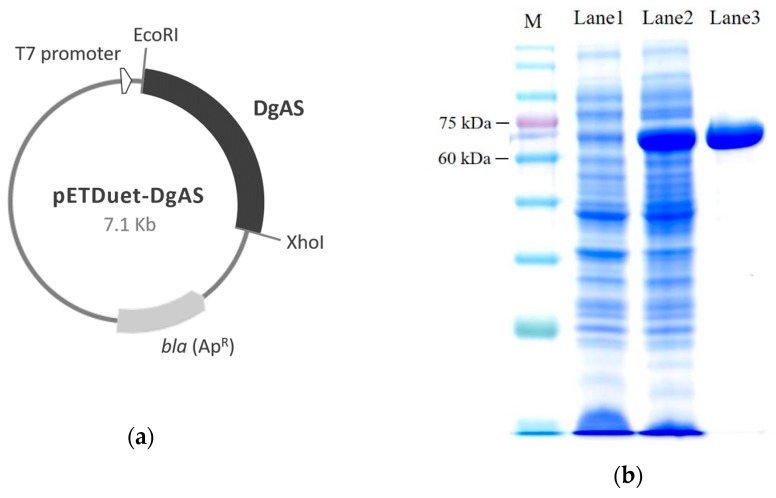
Production of the DgAS from *Deinococcus geothermalis* in *E. coli*. (**a**) Diagram of the constructed plasmid; (**b**) SDS-PAGE of the produced DgAS in recombinant *E. coli*. M: protein marker; lane 1: total protein without IPTG-induction; lane 2: total protein with IPTG-induction for 20 h; lane 3: purified DgAS.

The *DgAS* gene was amplified from genomic DNA of *Deinococcus geothermalis*, and subcloned into an expression plasmid *pETDuet-1* fused with six histidine residues in the N-terminal. The constructed *pETDuet-DgAS* plasmid (Figure 1a) was overexpressed in *E. coli*, and induced with 0.2 mM of isopropyl β-D-1-thiogalactopyranoside (IPTG). The soluble DgAS proteins were then successfully purified with Ni^2+^ chelate affinity chromatography, as shown in sodium dodecyl sulfate polyacrylamide gel electrophoresis (SDS-PAGE) (Figure 1b).

### 2.2. Biotransformation of 8-OHDe by Recombinant DgAS Protein

**Figure 2:**
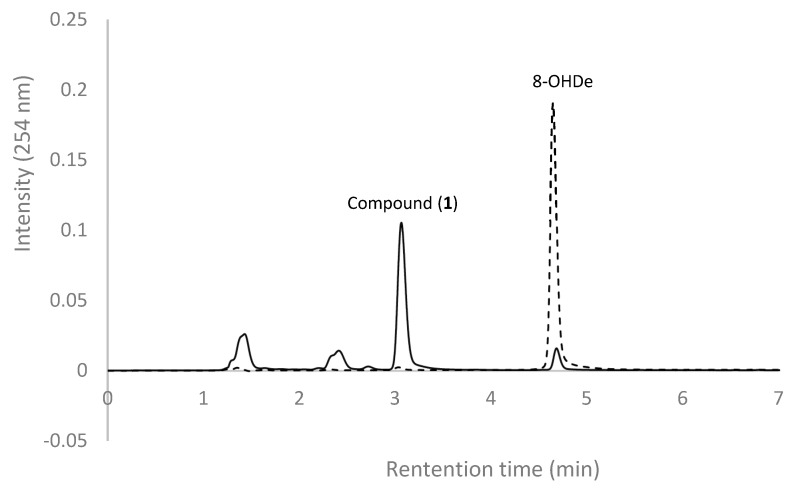
Biotransformation of 8-OHDe with DgAS. The reaction mixtures at 0 min (dashed line) and 30 min (solid line) were analyzed with UPLC. The UPLC operation conditions are described in Materials and Methods.

The purified DgAS were used to catalyze the biotransformation of 8-OHDe. The reaction contained 0.1 mg/mL of 8-OHDe, 12.5 µg/mL of DgAS, and 20 mM of sucrose as the sugar donor. The reaction mixtures at 0 min (the dashed line) and after 30-min incubation were analyzed with ultra-performance liquid chromatography (UPLC). One major product with a retention time (RT) of 3.1 min was observed (Figure 2).

**Figure 3:**
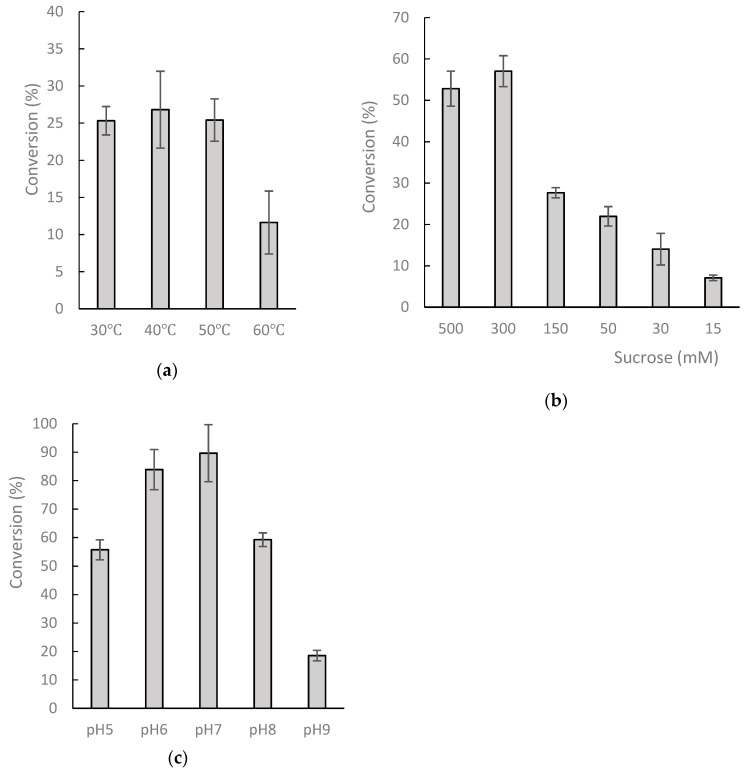
Optimal condition for the production of compound (**1**) from 8-OHDe by DgAS. (**a**) 125 µg of the purified DgAS and 1 mg/mL of 8-OHDe were incubated at different temperatures, (**b**) serial concentrations of sucrose, and (**c**) pH levels for 30 min. After incubation, the reaction was analyzed with UPLC. The conversion was calculated by dividing the amount of the produced compound (**1**) in each reaction by the theoretical production value (1.6 mg) for 100% conversion. The mean (*n* = 3) is shown, and the standard deviations are represented by error bars.

To optimize compound (**1**) production for further analysis, a standard mixture with 1 mg/mL of 8-OHDe and 125 µg/mL of DgAS was carried out at different temperatures, pH levels, and serial concentrations of sucrose for 30-min incubation. After incubation, the production conversion of the major product was determined with UPLC (Figure 3). The results revealed the optimal condition for the production of compound (**1**) from 8-OHDe by the recombinant DgAS is pH 7, 40 °C, and 300 mM of sucrose.

### 2.3. Identification of the Major Product

**Figure 4:**
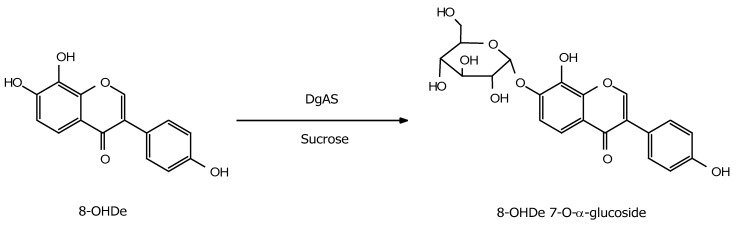
Biotransformation process of 8-OHDe by DgAS.

To resolve the chemical structure of the product, the biotransformation was scaled up to 100 mL, with 1 mg/mL of 8-OHDe, 125 µg/mL of DgAS, and 300 mM of sucrose at pH 7 and 40 °C for 30-min incubation. About 90 mg of the product in the 100-mL reaction was purified with preparative high-performance liquid chromatography (HPLC). Based on the value of the optimal conversion (89.3%) (Figure 3), the maximum production yield of compound (**1**) from 100 mg of 8-OHDe is 142.3 mg (160 mg × 0.89); thus, the purification recovery yield is 62.5% (90/142). The chemical structure of the purified compound (**1**) was resolved with mass and nuclear magnetic resonance (NMR) spectral analysis. The mass analysis of the compound showed an [M + H]^+^ ion peak at *m/z*: 433.18 in the electrospray ionization mass (ESI-MS) spectrum corresponding to the molecular formula C_21_H_20_O_10_. Then ^1^H and ^13^C NMR spectra, including distortionless enhancement by polarization transfer (DEPT), heteronuclear single quantum coherence (HSQC), heteronuclear multiple bond connectivity (HMBC), correlation spectroscopy (COSY), and nuclear Overhauser effect spectroscopy (NOESY) spectra, were obtained, and the ^1^H and ^13^C NMR signal assignments were conducted accordingly (shown in Figures S1–S7). In addition to the signals of 8-OHDe, seven proton signals (from 3.22 to 5.48 ppm) and six carbon signals (from 60–100 ppm) indicated a glucose moiety. The J coupling constant (3.6 Hz) of the anomeric proton (5.48 ppm) in the ^1^H NMR spectra of compound (1) indicated the α-configuration for the glucopyranosyl moiety. The cross peak of H-1″ with C-7 (5.48/148.8 ppm) in the HMBC spectrum, as well as the cross peak of H-1″ with H-6 (5.48/7.38 ppm) in the NOESY spectrum, demonstrated the structure of compound (**1**) was 8-OHDe-7-*O*-α-glucoside. The key HMBC and NOESY correlations of compound (**1**) are shown in Figure S8, and the spectroscopic data is listed in Table S1. The downfield shift of the ^1^H signal H-1″, the anomeric proton, of compound (**1**) compared to that of 8-OHDe-7-*O*-β-glucoside [20], indicated their different microenvironments. Figure 4 illustrates the biotransformation process of 8-OHDe by DgAS.

### 2.4. Solubility, Stability, and Anti-Inflammatory Activity of 8-OHDe 7-α-glucoside.

The aqueous solubility of 8-OHDe-7-*O*-α-glucoside was examined (Table 1). The results revealed that the maximum aqueous solubility of the 8-OHDe-7-*O*-α-glucoside is 3.92-fold, higher than that of 8-OHDe.

**Figure 5:**
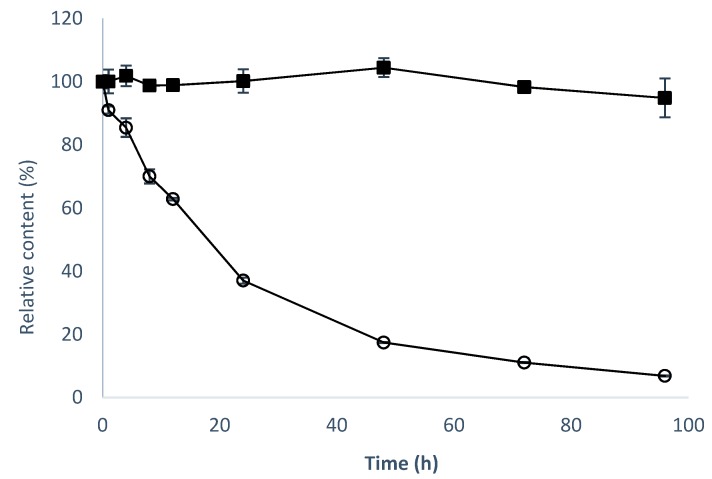
Alkaline stability of 8-OHDe (open circle) and 8-OHDe-7-*O*-α-glucoside (closed square). A total of 1 mg/mL of the tested compound was dissolved in 50 mM of Tris buffer at pH 8.0, and stored at 20 °C for 96 h. During the storage time, samples were taken out for UPLC at the determined interval times. The mean (*n* = 3) is shown, and the standard deviations are represented by error bars.

In addition, 8-OHDe is very unstable in the alkaline condition [19]. This property shortens the storage time of 8-OHDe in cosmetic or pharmaceutical products, and limits applications of 8-OHDe. Thus, the stability of the 8-OHDe-7-*O*-α-glucoside and 8-OHDe was compared (Figure 5). The half-time of 8-OHDe was 15.8 h, and only 6.8% of 8-OHDe remained in 50 mM of Tris buffer (pH 8.0) after 96-h incubation at 20 °C. However, 94.6% of 8-OHDe-7-*O*-α-glucoside still remained after 96-h incubation at the same condition. The half-time of 8-OHDe-7-*O*-α-glucoside was much longer than 96 h. Thus, 8-OHDe-7-*O*-α-glucoside is more than six-fold more stable than 8-OHDe in an alkaline solution.

**Figure 6:**
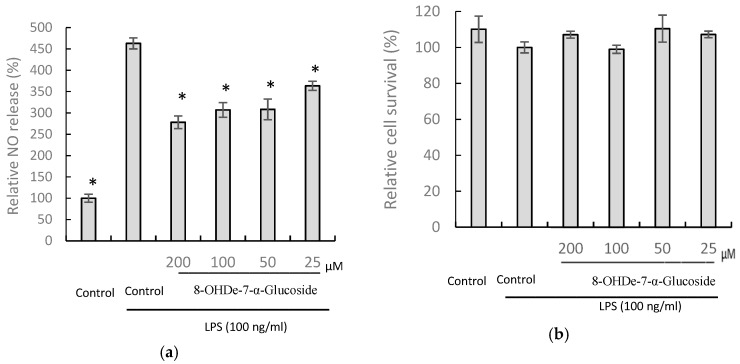

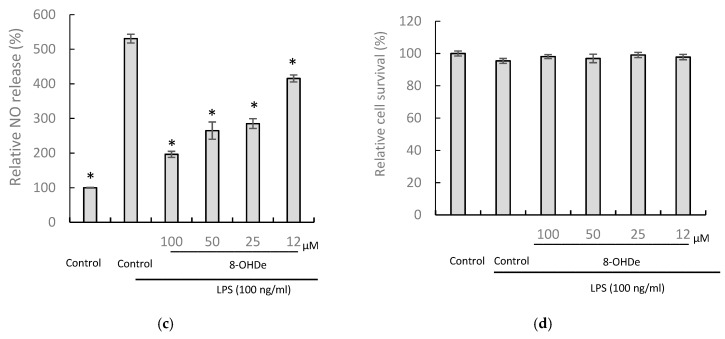
Effects of 8-OHDe-7-α-glucoside (**a**,**b**) and 8-OHDe (**c**,**d**) on the inhibition of LPS-induced NO production (**a**,**c**) and cell survival (**b**,**d**) in murine macrophage RAW264.7 cells. Cells were incubated with the indicated concentrations of isoflavone for 1 h before treatment with LPS (100 ng/mL) for 24 h. The amounts of NO were determined using the Griess reagent in the culture medium. Cell viability was determined with MTT assay. Each value indicates the mean ± standard deviation (SD), and is representative of the results obtained from four independent experiments. * (*p* < 0.001) is statistically significantly different from the value for the cells treated with LPS treatment alone.

Since the 8-OHDe-7-*O*-α-glucoside possesses much higher aqueous solubility and alkaline stability than those of 8-OHDe, and 8-OHDe was recently demonstrated with potent anti-inflammatory activity [15,16], the anti-inflammatory activity of 8-OHDe-7-*O*-α-glucoside and 8-OHDe was determined by the inhibition ability on lipopolysaccharide (LPS)-induced nitric oxide (NO) production in murine macrophage RAW264.7 cells. Macrophages are involved in chronic inflammation, and once macrophages are elicited, an inflammatory mediator, such as NO, is produced. Thus, the NO level in the culture supernatant could be measured as an index of inflammatory mediators. The results of the anti-inflammatory assays indicated 8-OHDe-7-α-glucoside exhibited statistically significant and dose-dependent inhibitory activity with an IC_50_ value of 173.2 ± 12.9 µM (Figure 6a), while 8-OHDe showed potent anti-inflammatory activity with an IC_50_ value of 34.5 ± 5.3 µM (Figure 6c). In addition, the results of cell survival assay indicated that 100 ng/mL of LPS treatment would not induce statistically significant cell death as the false-positive signal and reduction of NO by the tested isoflavones was not due to the cytotoxicity of the isoflavones (Figure 6b,d).

## 3. Discussion

Most GTs require additional expensive sugar donors, such as UDP-glucose, to generate glucose conjugates. In contrast, amylosucrase is particularly useful for glucosylation of flavonoids, due to its ability to use sucrose, an inexpensive and abundant renewable substrate, as a sugar donor. The studied amylosucrase (DgAS) was first identified by Stephane et al. [27], who expressed DgAS with N-terminal fusion of glutathione-S-transferase (GST) in *E. coli*, and found that the GST-DgAS was an inactive protein insoluble in the inclusion bodies. The N-terminal GST needed to be removed for the functional glucosylation activity. In contrast, Lee et al. recently produced recombinant DgAS in *E. coli* with fusion of His-tag in its C-terminal, and successfully purified the fusion DgAS as an active form with a simple Ni^2+^ affinity chromatography method [29]. The authors used the DgAS to catalyze glucosylation of hydroquinone forming α-arbutin, and their results showed an optimal reaction condition at pH 7, 40 °C, and 300 mM of sucrose. In the present study, we expressed DgAS with N-terminal His-tag fusion in *E. coli,* and successfully purified the fusion DgAS as an active form with a similar method (Figure 1 and Figure 2). After induction, 12.5 mg of well-soluble DgAS could be purified from 150 mL of the cell cultivation. Moreover, the optimal condition of major compound production by the DgAS enzyme (Figure 3) was consistent with that of Lee et al. [29], although we used 8-OHDe as the sugar acceptor. Thus, it seems that either N-terminal (the present study) or C-terminal [29] His-tag fusion is a more suitable strategy than GST fusion for DgAS production in *E. coli*.

Based on the naturally catalytic property of DgAS, which syntheses glucan polymer (amylose) from hydrolysis of the sucrose substrate, DgAS prefers catalyzing glucosylation sites at the hydroxyl group of an existing sugar moiety as the sugar acceptor [27,28]. Thus, it is easy to predict the catalytic products from the flavonoid glycosides substrate by DgAS, which would add a glucose moiety from sucrose to the hydroxyl group of sugar moiety in the flavonoid glycoside [30,31]. However, it is difficult to predict the *O*-glucosylation product when a flavonoid aglycone contains multiple hydroxyl groups as the sugar acceptor, due to the lack of sugar moiety in the structure. Thus far, DgAS has been reported to catalyze *O*-glucosylation only toward three flavonoid aglycones, catechin (5,7,3′,4′-tetrahydroxyflava-3-ol) [32], baicalein (5,6,7-trihydroxyflavone) [33], and 8-OHDe (7,8,4′-trihydroxyisoflavone, the present study), to form catechin-3′-*O*-α-glucoside, baicalein-6-*O*-α-glucoside, and 8-OHDe-7-*O*-α-glucoside, respectively. The three flavonoid aglycones contain a hydroxyl group at C7; however, only 8-OHDe was glucosylated at the C7 site by DgAS (Figure 4). It seems that DgAS prefers the two sites C3′ and C6 rather than C7 on flavonoid aglycones for glucosylation. The detail molecular mechanism for the glucosylation sites toward flavonoid aglycones by DgAS must be systematically studied in the future.

Drugs with poor aqueous solubility exhibit dilution rate-limited absorption in the membrane of the gastrointestinal tract; therefore, enhancing the solubility of drugs that have poor water solubility is an important issue in pharmaceutical research [32]. For example, although 8-OHDe is highly valuable in pharmaceutical research [3,4,5,6,7,8,9,10,11,12,13,14,15,16], its applications have been restricted due to its poor water solubility and alkaline instability in aqueous solution. Furthermore, the stability of flavonoids at various pH levels is also important for absorption in the gut, because of the sharp increase in pH from the acidic stomach to the slightly alkaline intestine. Glucosylation of flavonoids could improve such limitations. The stability and solubility of the glycosylated product were found to be drastically increased when compared to their aglycones [20]. In the present study, glucosylation significantly extended the half-life of 8-OHDe (Figure 5), and increased 3.92-fold the aqueous solubility of 8-OHDe (Table 1). Therefore, the glucosylated product, 8-OHDe-7-*O*-α-glucoside, has high potential in the pharmaceutical industry.

However, glucosylated flavonoids sometimes lose the bioactivity of their flavonoid precursors, although glucosylation could improve aqueous solubility, and extend the half-life of flavonoids. Since 8-OHDe has been shown to exhibit high anti-inflammatory activity [15,16], we wanted to determine the effect of glucosylated 8-OHDe on anti-inflammatory activity. As expected, the results showed that 8-OHDe-7-*O*-α-glucoside maintains only 20.1% of the anti-inflammatory activity of 8-OHDe (Figure 6), which is consistent with Hamalainen et al.’s results [34]. Hamalainen et al. also found inhibition of NO production in RAW264.7 cells by soy isoflavone genistein (97% of inhibition of NO production at the 100-µM concentration) was about five-fold higher than that of genistein-7-β-glucoside, genistin (17% of inhibition of NO production at the 100-µM concentration). The results reveal that the glucosyl group of the isoflavone skeleton would induce a negative effect on the exhibition of the anti-inflammatory activity. The detailed structure-activity relationship needs to be studied in the future. In addition, the signaling pathways involving in the anti-inflammatory activity by 8-OHDe in macrophage cells was already determined by Wu et al. [15] and Kim et al. [16], who demonstrated that the signals in both nuclear factor κB (NF- κB) and activator protein 1 (AP1) signaling pathways were inhibited by 8-OHDe in the anti-inflammation. Therefore, it is worthy to know if 8-OHDe-7-α-glucoside has similar signaling mechanism of anti-inflammation in the future. Nevertheless, the trade-off improved solubility, and long half-life alkaline-stability could extend the potential application of 8-OHDe-7-*O*-α-glucoside in anti-inflammation.

## 4. Materials and Methods

### 4.1. Microorganisms, Animal Cells, and Chemicals

*Deinococcus geothermalis* DSM11300 (BCRC17378) and mouse macrophage cells RAW 264.7 (BCRC60001) were obtained from the Bioresources Collection and Research Center (BCRC, Food Industry Research and Development Institute, Hsinchu, Taiwan), and cultivated according to the BCRC protocol. *E. coli* BL21 (DE3) and the *pET-Duet-1* expression plasmid were obtained from the Novagen Company (Madison, WI, USA). Restriction enzymes and DNA modified enzymes were obtained from New England Biolabs (Ipswich, MA, USA). 8-OHDe was prepared according to Dr. Wu’s method [18]. IPTG, 3-(4,5-dimethylthiazol-2-yl)-2,5-diphenyltetrazolium bromide (MTT), dimethyl sulfoxide (DMSO), LPS, and Greiss reagent were purchased from Sigma (St. Louis, MO, USA). The other reagents and solvents used are commercially available.

### 4.2. Preparation of Recombinant DgAS Enzyme

*DgAS* was amplified from the genome of *Deinococcus geothermalis* with polymerase chain reaction (PCR) with a primer set: forward 5′-cccgaattcgCTGAAAGACGTGCTCACTTCTGAAC-3′ and reverse 5′-aaactcgagTTATGCTGGAGCCTCCCCGGCGGTC-3′, which contained EcoRI and XhoI restriction sites, respectively. The amplified DNA fragment (1.95 kb) was digested with EcoRI and XhoI, and ligated into the corresponding sites on the *pET-Duet-1* expression plasmid to form *pETDuet-DgAS* (Figure 1a), which was then transformed into *E. coli* (DE3) with the electroporation method [20]. The recombinant *E. coli* (DE3) was cultured in Luria-Bertani (LB) medium to optical density at 560 nm (OD_560_) of 0.6, and then induced with 0.2 mM of IPTG. After further cultivation at 18 °C for 20 h, the cells were centrifuged at 4500× *g* and 4 °C for 20 min. The cell pellet was washed, and spun down twice with 50 mM of phosphate buffer (PB) at pH 6.8, and then broken with sonication via a Branson S-450D Sonifier (Branson Ultrasonic Corp., Danbury, CT, USA). The sonication program was carried out for five cycles of 5 sec on and 30 sec off at 4 °C. The mixture was then centrifuged at 15,000× *g* and 4 °C for 20 min to remove the cell debris. The supernatant containing the produced DgAS fused with a His-tag in its N-terminal was applied in a Ni^2+^ affinity column (10 i.d. × 50 mm, Ni Sepharose 6 Fast Flow, GE Healthcare, Chicago, IL, USA). The His-tag fused DgAS was washed with PB with 25 mM of imidazole and eluted with PB containing 250 mM of imidazole. The elution was then concentrated and desalted through Macrosep 10 K centrifugal filters (Pall, Ann Arbor, MI, USA). The concentration of the purified DgAS was determined with the Bradford method [20], and analyzed with SDS-PAGE (Figure 1b). The purified DgAS was stored in a final concentration of 50% glycerol at –80 °C before use.

### 4.3. Biotransformation of 8-OHDe by the Purified DgAS Enzyme

Biotransformation was carried out in 1 mL of reaction mixture containing 0.1 mg/mL of 8-OHDe, 12.5 µg/mL of DgAS, 150 mM of sucrose, and 50 mM of Tris, pH 8.0. The reaction was performed at 40 °C for 30 min. After reaction, the reaction mixture was analyzed with UPLC. To optimize the major compound production, 1 mg/mL of 8-OHDe and 125 µg of DgAS were used as the substrate at different temperatures, pH levels, and sucrose concentrations. To optimize the pH, 50 mM of acetate buffer (pH 5 and pH 6), phosphate buffer (pH 7), and Tris buffer (pH 8 and pH 9) were used.

### 4.4. UPLC

UPLC was performed with an Acquity^®^ UPLC system (Waters, Milford, MA, USA). The stationary phase was the Kinetex^®^ C18 column (1.7 µm, 2.1 i.d. × 100 mm, Phenomenex Inc., Torrance, CA, USA), and the mobile phase was 1% acetic acid in water (A) and methanol (B). The linear gradient elution condition was 0 min with 36% B to 7 min with 81% B at a flow rate of 0.2 mL/min. The detection condition was set at 254 nm.

### 4.5. Purification and Identification of the Biotransformation Product

One hundred milliliters of the reaction mixture (1 mg/mL of 8-OHDe, 125 µg/mL of DgAS, 300 mM of sucrose, 50 mM of phosphate buffer, pH 7) was carried out at 40 °C for 30 min. At the end of the reaction, equal volume of methanol was added into the reaction mixture to stop the reaction. Two hundred milliliters of the reaction mixture with 50% of methanol was applied in a preparative YL9100 HPLC system (YoungLin, Gyeonggi-do, Korea). The stationary phase was the Inertsil ODS 3 column (10 mm, 20 i.d. × 250 mm, GL Sciences, Eindhoven, The Netherlands), and the mobile phase was the same as those in the UPLC system, but with a flow rate of 15 mL/min. The detection condition was 254 nm, and the sample volume was 10 mL for one injection. The product from each run was collected, concentrated under vacuum, and lyophilized with a freeze dryer. From the 100 mL of reaction, 90 mg of the major compound was purified. The chemical structure of the major compound was determined with mass and NMR spectral analysis. The mass spectral analysis was performed on a Finnigan LCQ Duo mass spectrometer (ThermoQuest Corp., San Jose, CA, USA) with ESI. ^1^H- and ^13^C-NMR, DEPT, HSQC, HMBC, COSY, and NOESY spectra were recorded on a Bruker AV-700 NMR spectrometer (Bruker Corp., Billerica, MA, USA) at ambient temperature. Standard pulse sequences and parameters were used for the NMR experiments, and all chemical shifts were reported in parts per million (ppm, δ).

### 4.6. Determination of Aqueous Solubility and Stability

Aqueous solubility and stability were conducted with the methods we used in a previous study [20]. For aqueous solubility assay, the tested compound was vortexed in distilled deionized H_2_O for 1 h at 25 °C. The mixture was analyzed with UPLC. For stability assay, a stock of the tested compound (100 mg/mL in dimethyl sulfoxide) was diluted 100-fold to a concentration of 1 mg/mL in 50 mM of Tris buffer at pH 8.0. Then, samples were taken out for the UPLC analysis at the determined interval times.

### 4.7. Determination of Anti-Inflammatory Activity

The murine macrophage RAW 264.7 cell line was maintained in Dulbecco’s Modified Eagle Medium (DMEM) supplemented with 10% fetal bovine serum (FBS), 100 μg/L streptomycin, and 100 IU/mL penicillin at 37 °C in a 5% CO_2_ atmosphere. The RAW 264.7 cells were seeded at a density of 5 × 10^5^ cells/well in 24-well plates, and incubated for 12 h at 37 °C and 5% CO_2_. Different concentrations of the tested isoflavones were added. After 1-h treatment, the cells were stimulated with 100 ng/mL of LPS for 24 h. Culture supernatants (equal volumes) were mixed with Greiss reagent at room temperature for 10 min, and then the absorbance was measured at the wavelength 540 nm using a microplate reader (Sunrise, Tecan, Männedorf, Switzerland), as described previously [15]. This analysis was performed in tetraplicate. Relative inhibition of NO production was calculated with the equation: Relative inhibition (%) = [(OD_570_ with LPS only–OD_570_ with both LPS and isoflavones)/(OD_570_ with LPS only–OD_570_ without LPS or isoflavone)] × 100%. An IC_50_ value means a concentration of the drug that exhibited 50% of inhibition.

## 5. Conclusions

8-OHDe-7-α-glucoside is successfully produced from *O*-glucosylation of 8-OHDe with recombinant DgAS of *Deinococcus geothermalis* with a cheap and abundant renewable substrate, sucrose, as a sugar donor. The isoflavone glucoside is more soluble and stable than those of 8-OHDe in working buffers. The long half-life of 8-OHDe-7-α-glucoside maintains moderate anti-inflammatory activity, and could be used for industrial applications in the future.

## Figures and Tables

**Table 1:** Aqueous solubility of 8-OHDe and 8-OHDe-7-*O*-α-glucoside.

Compound	Aqueous-Solubility (mg/L)	Fold
8-OHDe	47.3	1
8-OHDe-7-*O*-α-glucoside	185.4	3.92

## Supplementary Materials

The following are available online at https://www.mdpi.com/1420-3049/24/12/2236/s1, Table S1. NMR spectroscopic data for compound (**1**) (in DMSO-d6; 700 MHz); Figure S1. The ^1^H-NMR (700 MHz, DMSO-d6) spectrum of compound (**1**); Figure S2. The ^13^C-NMR (176 MHz, DMSO-d6) spectrum of compound (**1**); Figure S3. The DEPT-90 and DEPT-135 (176 MHz, DMSO-d6) spectra of compound (**1**); Figure S4. The HSQC (700 MHz, DMSO-d6) spectrum of compound (**1**); Figure S5. The HMBC (700 MHz, DMSO-d6) spectrum of compound (**1**); Figure S6. The H-H COSY (700 MHz, DMSO-d6) spectrum of compound (**1**); Figure S7. The H-H NOESY (700 MHz, DMSO-d6) spectrum of compound (**1**); Figure S8. The key HMBC (blue arrows) and NOESY (pink arrow) correlations of compound (**1**).
