# Supplementary material for: Potential Industrial Production of a Well-Soluble, Alkaline-Stable, and Anti-Inflammatory Isoflavone Glucoside from 8-Hydroxydaidzein Glucosylated by Recombinant Amylosucrase of Deinococcus geothermalis
Source: Molecules. 2019 Jun 15;24(12):2236. doi: 10.3390/molecules24122236 (PMC6631725; doi:10.3390/molecules24122236)
Supplement: Supplementary file 1 [file molecules-24-02236-s001.pdf]

Potential Industrial Production of a Well-Soluble, Alkaline-Stable, and Anti-Inflammatory Isoflavone Glucoside from 8-Hydroxydaidzein Glucosylated by Recombinant Amylosucrase of *Deinococcus geothermalis*

Te-Sheng Chang [1,†,*], Tzi-Yuan Wang [2,†], Szu-Yi Yang [1], Yu-Han Kao [1], Jiumn-Yih Wu [3,*] and Chien-Min Chiang [4,*]

[1] Department of Biological Sciences and Technology, National University of Tainan, Tainan 70005, Taiwan; szuyi08231995@gmail.com (S.-Y.Y.); aa0920281529@gmail.com (Y.-H.K.)
[2] Biodiversity Research Center, Academia Sinica, Taipei 115, Taiwan; tziyuan@gmail.com
[3] Department of Food Science, National Quemoy University, Kinmen County 892, Taiwan
[4] Department of Biotechnology, Chia Nan University of Pharmacy and Science, No. 60, Sec. 1, Erh-Jen Rd., Jen-Te District, Tainan 71710, Taiwan
* Correspondence: mozyme2001@gmail.com (T.-S.C.); wujy@nqu.edu.tw (J.-Y.W.); cmchiang@mail.cnu.edu.tw (C.-M.C.); Tel.: +886-6-2606283 (T-S. C.); +886-82-313310 (J.-Y.W.); +886-6-2664911 ext. 2542 (C.-M.C.); Fax: +886-6-2606153 (T.-S.C.); +886-82-313797 (J.-Y.W.); +886-6-2662135 (C.-M.C.)
† These authors contributed equally to this work.

Table S1. NMR spectroscopic data for compound (**1**) (in DMSO-d_6; 700MHz).

Compound	8-OHDe-7-*O*- -glucoside Compound (**1**)		-	8-OHDe [a]		8-OHDe-8-*O*- - glucoside [b]	
Position	C	H (*J* in Hz)	HMBC	C	H (*J* in Hz)	C	H (*J* in Hz)
Isoflavone moiety							
2	153.7	8.40, s		153.0	8.30, s	153.0	8.32, s
3	123.5		H-2, H-2′(6′), H-3′(5′)	123.0		123.3	
4	175.8		H-2, H-5	175.6		175.0	
5	115.0	7.51, d (8.9)		116.0	7.45, d (8.7)	121.5	7.76, d (8.8)
6	114.6	7.38, d (8.9)	H-5,	114.5	6.94, d (8.7)	115.5	7.02, d (8.8)
7	148.8		H-5, H-6 [c]	150.2	9.46,br s, OH [d]	154.6	
8	136.6		H-5, H-6	133.2	10.37,br s,OH [d]	131.9	
8a	146.4		H-2, H-5, H-6	147.0		150.6	
4a	120.1		H-6,	117.7		117.4	
1′	122.9		H-3′(5′)	123.2		122.6	
2′(6′)	130.6	7.40, m	H-2′(6′), H-3′(5′)	130.4	7.37, m	130.2	7.38, m
3′(5′)	115.5	6.82, m	H-2′(6′), H-3′(5′)	115.3	6.81, m	115.1	6.81, m
4′	157.6	-	H-2′(6′), H-3′(5′)	157.4	-	157.3	-
		9.67 OH [d]			9.58, br s OH [d]		9.63 OH [d]
Glucose moiety							
1′′	100.0	5.48, d (3.6)				104.5	4.93, d (7.7)
2′′	72.3	3.41, m	H-3′′			74.1	3.36, m
3′′	73.5	3.75, m	H-1′′, H-2′′, H-4′′			76.4	3.27, m
		5.09 OH[d]					5.21 OH [d]
4′′	70.2	3.22, m	H-3′′, H-5′′, H-6′′			69.8	3.18, m
		5.13 OH[d]					5.07 OH [d]
5′′	74.4	3.46, m	H-1′′, H-4′′, H-6′′			77.4	3.18, m
6′′	61.0	3.61, m	H-4′′, H-5′′			60.9	3.63, m
		3.51, m					3.47, m
		4.64 OH [d]					4.50 OH [d]

(a) Chang T.-S.; Ding, H.-Y.; Tai, S.-K.; Wu, C.-Y. Mushroom tyrosinase inhibitory effects of isoflavones isolated from soygerm koji fermented with *Aspergillus oryzae* BCRC 32288. *Food Chemistry*. **2007**, 105, 1430–1438.

(b) Chiang, C.-M.; Wang, T.-Y.; Yang, S.-Y.; Wu, J.-Y.; Chang, T.-S. Production of New Isoflavone Glucosides from Glycosylation of 8-Hydroxydaidzein by Glycosyltransferase from *Bacillus subtilis* ATCC 6633. *Catalysts*. **2018**, *8*, 387.

(c) A correlation between Glc-H-1′′ and C-7 was observed in HMBC spectrum of compound (1).

(d) OH indicates the hydroxy proton.

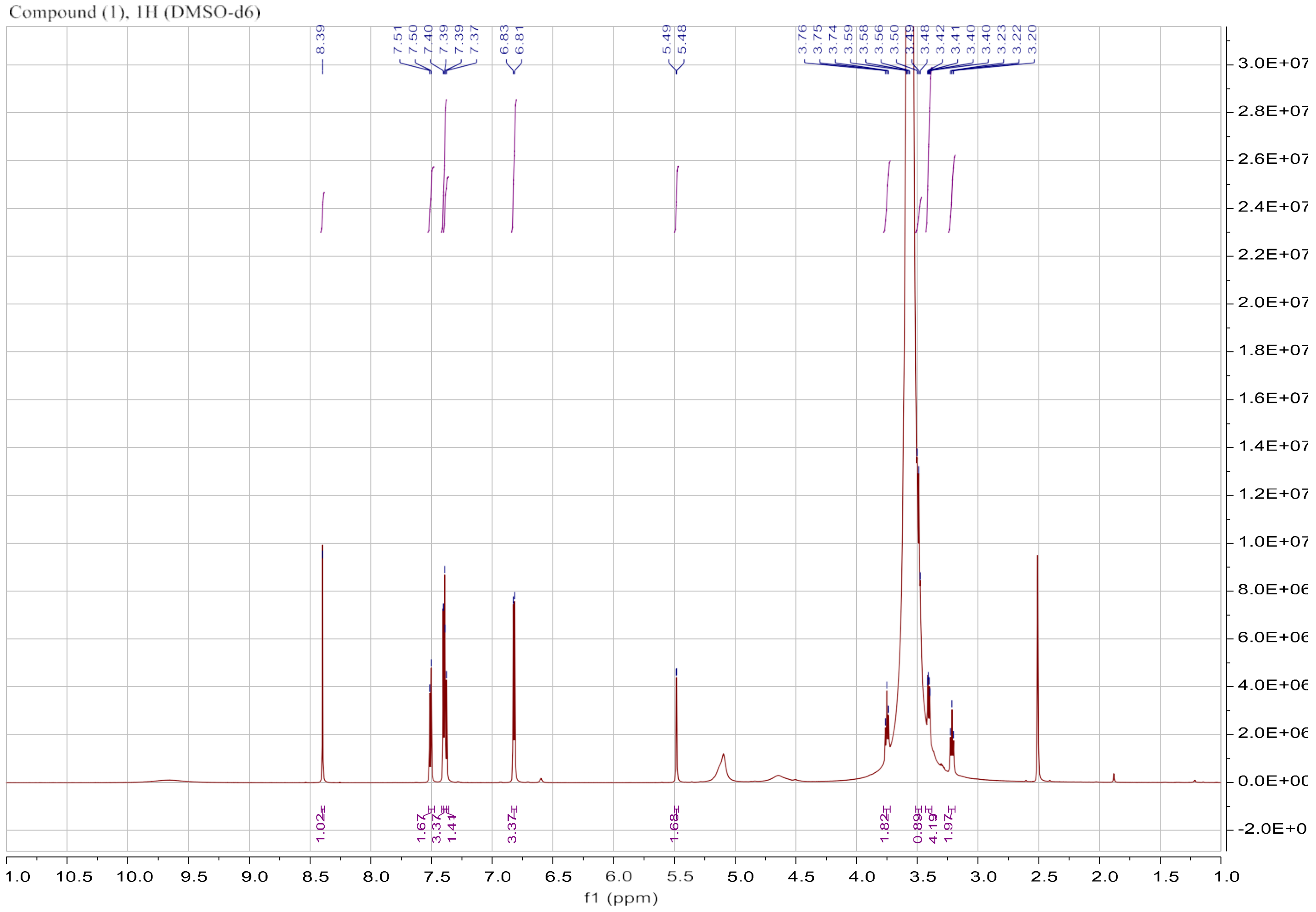

Figure S1. The ^{1}H NMR (700 MHz, DMSO-*d6*) spectrum of compound (**1**).

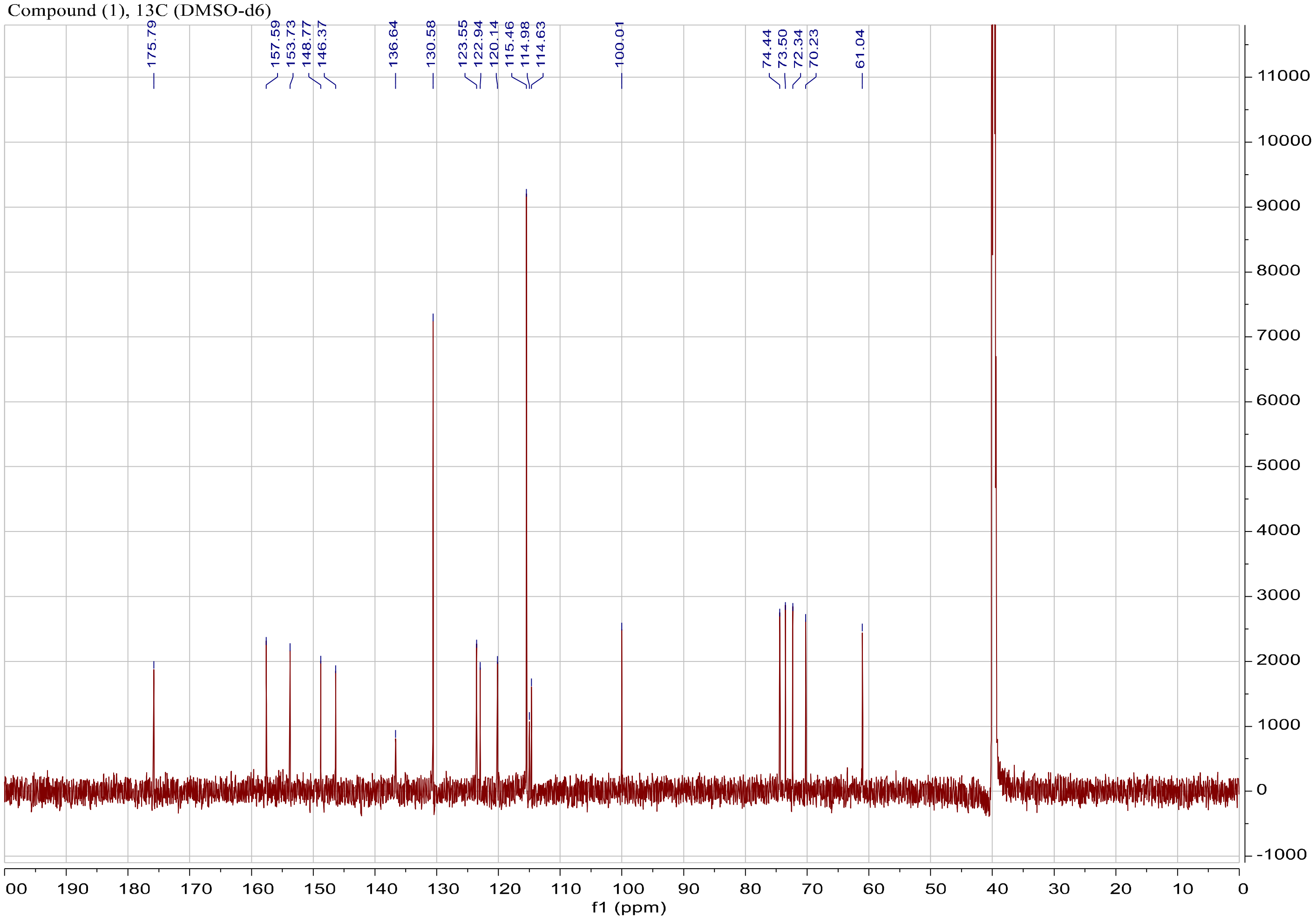

Figure S2. The ^{13}C NMR (176 MHz, DMSO-*d*6) spectrum of compound (**1**).

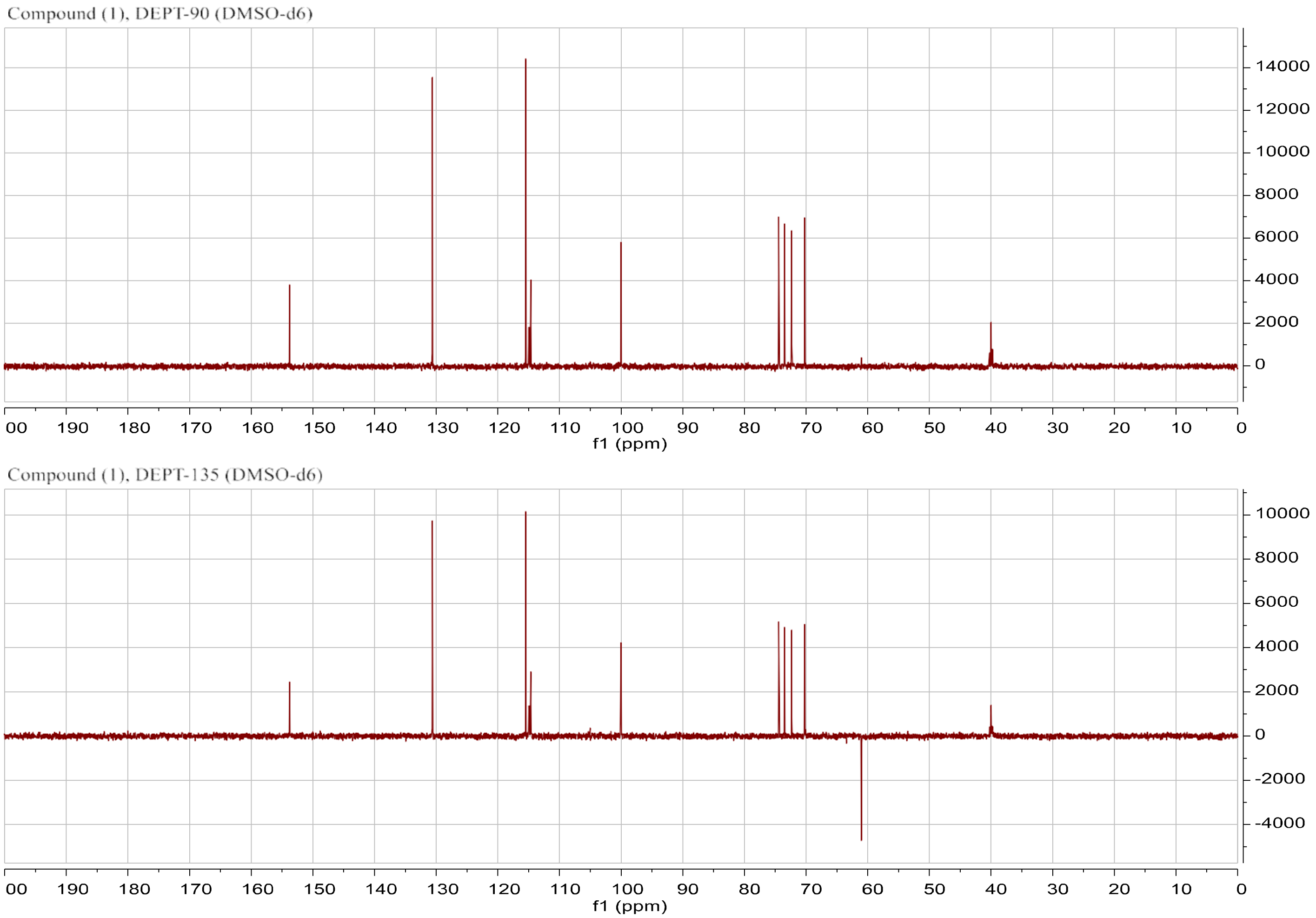

Figure S3. The DEPT-90 and DEPT-135 (176 MHz, DMSO-*d6*) spectra of compound (**1**).

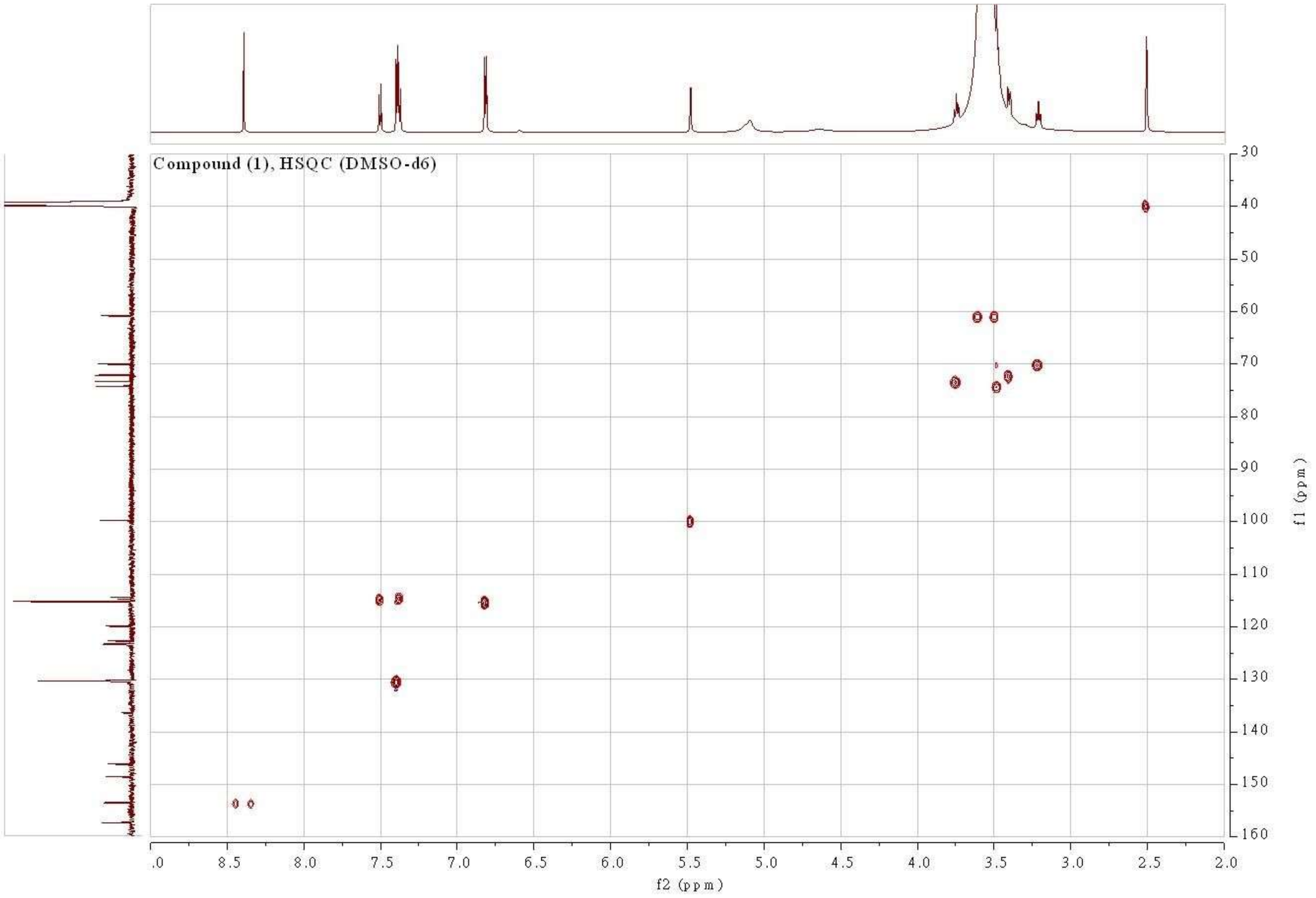

Figure S4. The HSQC (700 MHz, DMSO-*d6*) spectrum of compound (**1**).

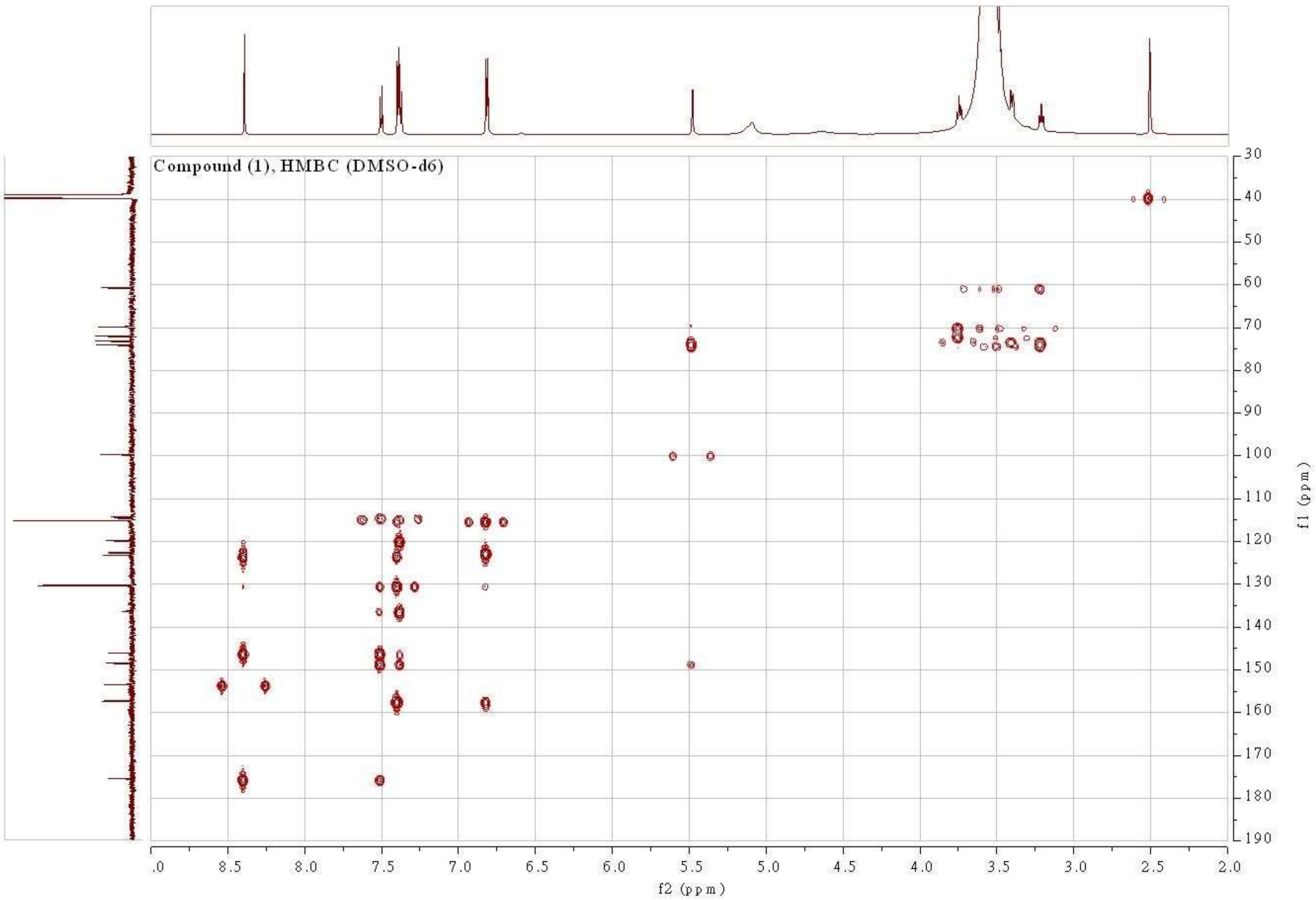

Figure S5. The HMBC (700 MHz, DMSO-*d*6) spectrum of compound (**1**).

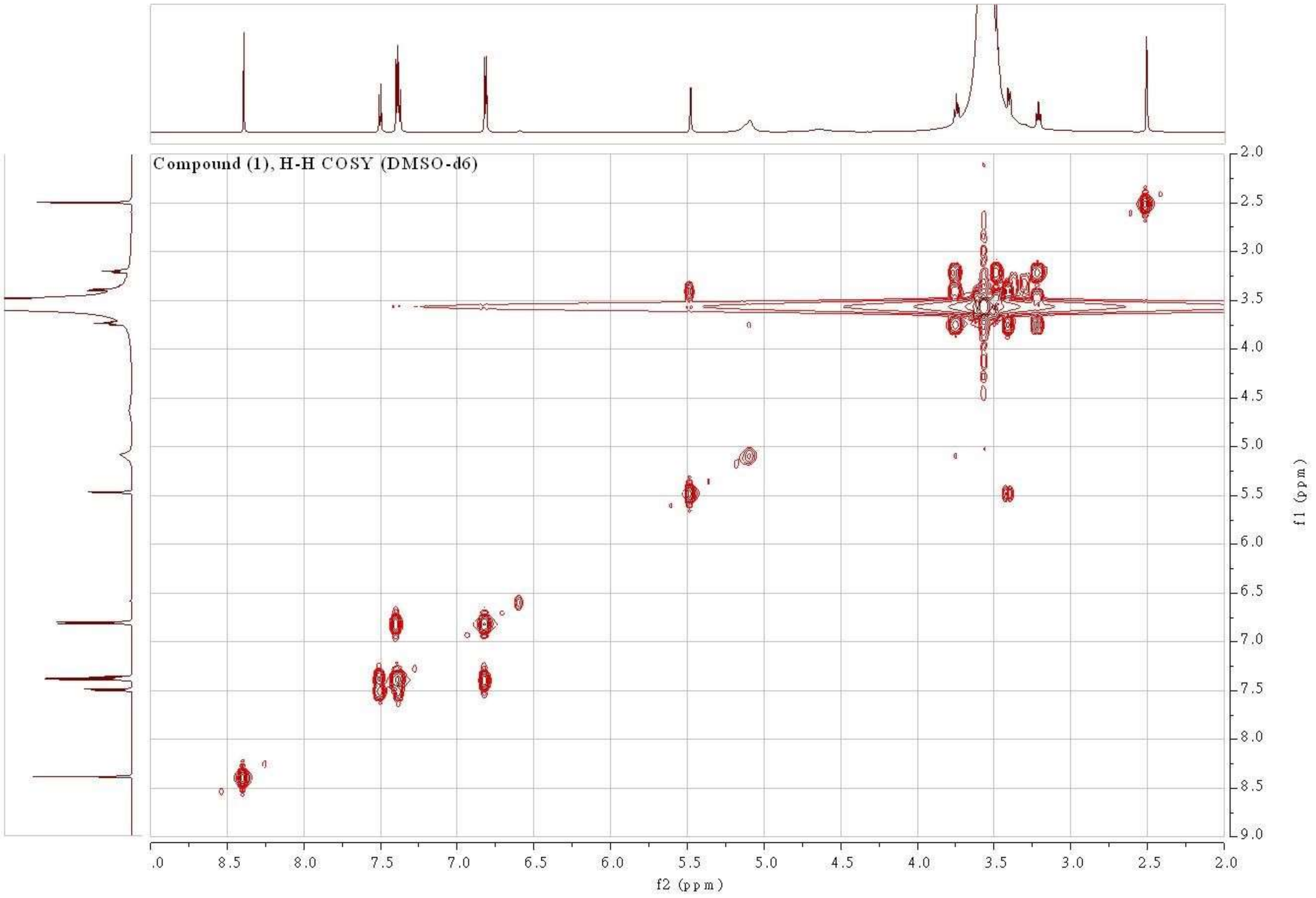

Figure S6. The H-H COSY (700 MHz, DMSO-*d6*) spectrum of compound (**1**).

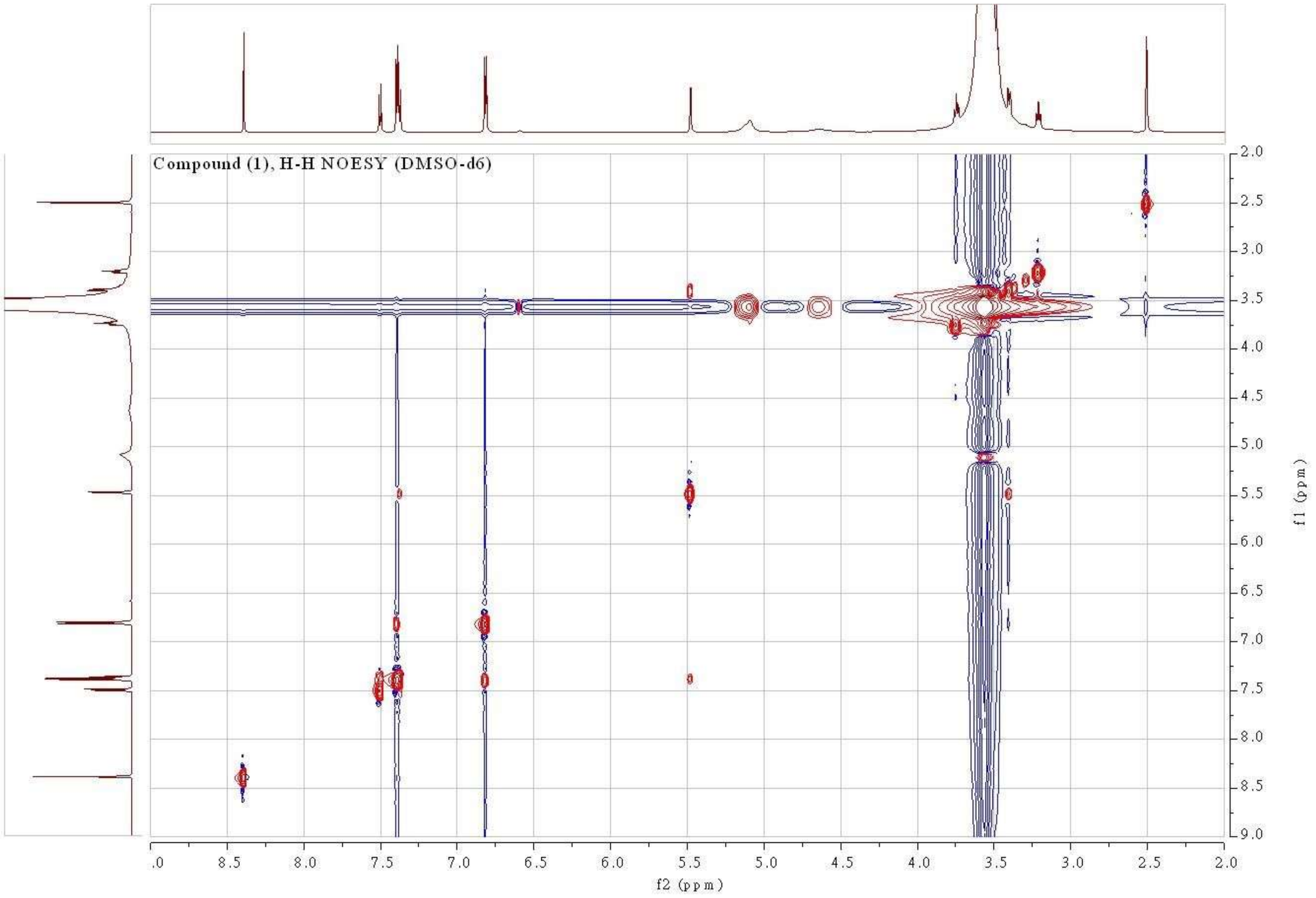

Figure S7. The H-H NOESY (700 MHz, DMSO-*d6*) spectrum of compound (**1**).

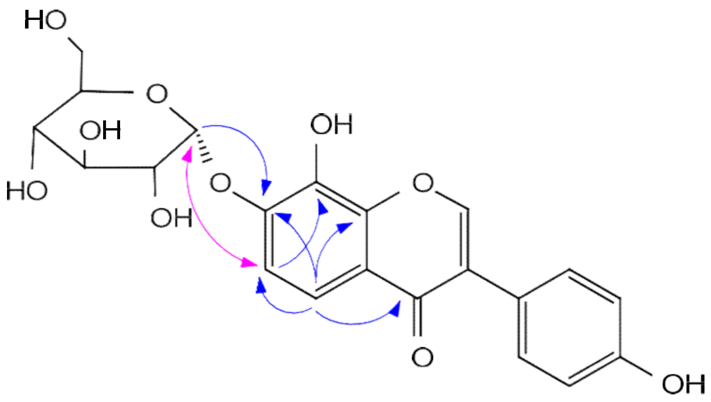

Figure S8. The key HMBC (blue arrows) and NOESY (pink arrow) correlations of compound (**1**).
